# *HspB5* correlates with poor prognosis in colorectal cancer and prompts epithelial-mesenchymal transition through ERK signaling

**DOI:** 10.1371/journal.pone.0182588

**Published:** 2017-08-10

**Authors:** Qinghua Li, Yanlan Wang, Yuexing Lai, Ping Xu, Zhiwen Yang

**Affiliations:** 1 Songjiang Hospital Affiliated Shanghai First People’s Hospital, Shanghai Jiao Tong University, Shanghai, China; 2 Shanghai Songjiang Hospital Affiliated to Nanjing Medical University, Nanjing, China; University of South Alabama Mitchell Cancer Institute, UNITED STATES

## Abstract

Alpha B-crystallin (*HspB5*) is abnormally expressed in tumor tissues and portends a poor prognosis in cancer patients. However, the role of *HspB5* in colorectal cancer (CRC) is still unclear. Seventy CRC patients and 40 healthy volunteers were sampled from August 2012 to March 2015 in order to determine the clinical significance of *HspB5*. *In vitro* cellular studies were used to validate its molecular mechanisms in CRC. Our clinical data indicated that *HspB5* was up-regulated, and had a positive association with TNM stage CRC patients. The expression level of *HspB5* in CRC patients was closely correlated with MMP7 and E-cadherin, two core epithelial–mesenchymal transition (EMT) gene products. The *in vitro* studies revealed that high *HspB5* expression could prompt tumor cell proliferation and invasion, as well as EMT. Gene-microarray analysis suggested three significant signaling pathways (PI3K, p38 and ERK) were involved in *HspB5*-induced EMT. Signal transduction pathway inhibitors and *HspB5* gene knockdown models suggested that *HspB5* promotes CRC tumorigenesis and EMT progression through ERK signaling pathways. In summary, *HspB5* maybe trigger the EMT in CRC by activating the ERK signaling pathway. It is a potential tumor biomarker for CRC diagnosis and prognosis.

## Introduction

Colorectal cancer (CRC) is the third most prevalent cancer in China and accounts for the major cause of cancer-related deaths[1]. As we know, the occurrence and progression of CRC maybe a multi-step, complex process associated with multiple oncogenes and tumor suppressors[2]. Although great efforts have been made to better understand the molecular mechanism relevant to CRC and to improve its therapy, these aspects of CRC are still largely unknown. The underlying pathogenesis associated with CRC requires further study for the development of novel diagnostic and therapeutic approaches.

Alpha B-crystallin (*HspB5*) is a cytoprotective molecular chaperone and small heat-shock protein[3]. It prevents the stress-induced, irreversible aggregation of denatured proteins, and traps aggregation-prone proteins in reservoirs of nonnative, and refoldable intermediates, within soluble, large, and multimeric structures[4]. Thus, *HspB5* is suggested to play a key role in cellular protection, apoptosis inhibition, and proteasomal interactions. Recent studies mainly focus on the presence of *HspB5* in various types of solid tumors as a novel oncoprotein, as well as a prognostic marker[5, 6]. For example, *HspB5* over-expression has been clinically detected in several different types of tumors including breast, renal, lung, and hepatic cancer, and is associated with poor prognosis in most of these patients[7–10]. However, little is known about the role and mechanism of *HspB5* in CRC.

CRC is a malignant tumor that is characterized by a high potential for invasion and metastasis[11]. It is widely accepted that epithelial–mesenchymal transition (EMT) acts as a fundamental mechanism in tumor invasion and metastasis, especially in CRC[12]. EMT is a process by which epithelial cells lose epithelial properties to become mesenchymal stem cells. Carcinoma cells undergoing EMT in primary tumors lose their cell polarity and cell to cell adhesion capacity, gain invasive properties[13], enter the bloodstream, and then contribute to clonal outgrowth at these metastatic sites[14]. Related studies are of great importance in determining whether *HspB5* expression facilitates tumor progression in CRC by inducing EMT.

In the present study, it was demonstrated that *HspB5* could be identified as a valuable biomarker for clinicopathological parameters and poor prognosis in CRC patients. The potential molecular mechanisms by which *HspB5*-induced EMT may be responsible for tumor invasion and metastasis was then explored. Taken together, this is the first report showing the clinical significance and molecular mechanism of *HspB5* in CRC.

## Materials and methods

### Cell lines

Three CRC cell lines, including HCT116, SW480and Lovo, were purchased from the American Type Culture Collection (Manassas, VA, USA). All cells were maintained in RPMI 1640 media supplemented with 10% fetal bovine serum under a 5% CO_2_ incubator at 37°C.

### Patients and follow-up

Fresh CRC tissue and adjacent nontumor tissue was collected from 70 consecutive patients who underwent colorectal surgery between August2012 and March 2015 at Songjiang Hospital Affiliated Shanghai First People’s Hospital. CRC differentiation was defined according to Dukes’grading system described by the World Health Organization. None of the patients received chemotherapy, radiotherapy or other special treatment before surgery. Forty normal colonic samples were obtained via colonoscopy from healthy people who underwent a health check in Songjiang Hospital Affiliated Shanghai First People’s Hospital. Follow-ups were terminated on May 15, 2016, following the procedures described in this paper[15, 16].

### Gene expression profile chip

Six human colonic tumors (N = 6) and their adjacent nontumor tissue (N = 6), as well as four normal intestinal mucosa tissue samples (N = 4) were included and subjected to gene expression microarray in the study. Subsequently, human gene microarray hybridization was performed according to the manufacturer’s instructions (Agilent Technologies, Santa Clara, CA, USA). Briefly, hybridization run conditions were as follows: speed (10 rpm), run time (up to 17 h), temperature (65°C). After the reaction was complete, the hybridization chip was washed with Gene Expression Wash Buffer Kit in wash tank staining dishes (Thermo Shandon, Waltham, MA, USA). After generating the microarray scan images through the Agilent Microarray Scanner (Agilent Technologies), the data was extracted using Feature Extraction software 10.7 (Agilent Technologies). The data were normalized using the Quantile algorithm, Gene Spring Software 11.0 (Agilent Technologies). Finally, the obtained data was exported directly into the SAM software for SAM analysis. Genes were considered to be differentially expressed based on the criteria of P<0.005, fc2, and mean7.

### RNA extraction and qRT-PCR

Seventy human colonic tumors, their adjacent nontumor tissue, and 40 normal intestinal mucosa samples were analyzed via qRT-PCR (Table 1). Total RNA of pancreatic tissue was isolated with a Trizol reagent (Invitrogen, CA, USA) according to the manufacturer’s instructions. The total RNA was treated with RNase-free DNase to remove the residual genomic DNA. 1 μg of total RNA was reverse-transcribed to the first-strand cDNA that was used as template for PCR amplification. The thermal amplification step was conducted in the following conditions: 95°C denaturation for 30s followed by 40 cycles of 95°C denaturation for 5s, and 60°C for 30s. In addition, HCT116, SW480 and Lovo cell lines were also analyzed via qRT-PCR as described previously.

**Table 1:** qRT-PCR using the following primers.

Gene	Forward(5'to3')	Reverse(5'to3')
*E-cadherin*	CACCACGTACAAGGGTCAGG	AACAGCTGTGAGGATGCCAG
*MMP7*	CTACAGTGGGAACAGGCTCA	CACTCCACATCTGGGCTTCT
*HspB5*	TTCCCGACGTCTACTTCCCT	TCCTGGCGCTCTTCATGTTT

### Immunofluorescence

CRC patient samples were subjected to immunofluorescence analysis. A monoclonal, anti-human *HspB5* antibody (Santa Cruz, CA, USA) was used to detect the expression of *HspB5* in colonic tissue samples. Paraffin-embedded tissue sections of colorectal cancers were stained as follows: the samples were incubated with a polyclonal anti-*HspB5* antibody overnight at 4°C, and then with a secondary antibody for 60 min at 37°C.

### Cell transfection

Small interfering RNAs (siRNA) were constructed and synthesized by Shanghai GenePharma Co, Ltd (Shanghai, China). Lipofectamine 2000 was purchased from Invitrogen Life Technologies (Carlsbad,CA, USA). Control siRNA and siRNA-*HspB5* were transfected into Lovo cells using Lipofectamine, according to the manufacturer’s instructions. Briefly, 20pmol *HspB5* siRNA or control siRNA was incubated with Lovo cells for 6h. The interference efficiency of the *HspB5* gene was defined using qRT-PCR and western blotting. The siRNA target sequences are listed below.

siRNA sequences: 5’-CCAUCACCCGUAGAAGAGAATT-3’;
Antisense:5’-UUCUCUUCACGGGUGAUGGTT-3’;
Control RNA sequences: 5’-UUCUCCGAACGUGUCACGUTT-3’;
Antisense:5’-ACGUGACACGUUCGGAGAATT-3’.

### Western blot

Western blotting was used to investigate *HspB5*, MMP-7 and E-cadherin protein expression in Lovo cells. Briefly, the samples were incubated overnight at 4°Cwith a polyclonal anti-*HspB5* antibody (Santa Cruz, CA,USA), an anti-MMP7 antibody (Santa Cruz, CA,USA), and an anti-E-cadherin antibody (Abcam, Cambridge, MA, USA). This was followed by incubation with a secondary antibody for 60 min at 37°C. Finally, western blot bands were captured though a gel imaging system (Bio-Rad, Hercules, CA, USA).

### Invasion and metastasis assays

Transwell migration assays (pore size, 8 μM) were performed to analyze tumor invasion and metastasis. Lovo cells were selected and respectively co-cultured with siRNAs, Ly294002 (25μM final concentration, PI3K inhibitor, Calbiochem, Billerica, MA, USA), SB202190 (5μM final concentration, p38 inhibitor, Calbiochem, Billerica, MA, USA), and PD98039 (20μM final concentration, ERK inhibitor, Calbiochem, Billerica, MA, USA). Briefly, cells were loaded on the upper compartment of the six-well Transwell chamber (pore size 8 μm; Dow Corning Corp, Burlington, NJ, USA), and allowed to migrate across an 8.0μm pore size polycarbonate membrane for 24 h. As a result of the chemo-attractant properties, cells migrated to the bottom surface of the membrane and were then counted to evaluate their invasion and metastasis abilities.

### Cell proliferation assay

Lovo cells were added to a 96-well plate at 1×10^4^/100μl/well concentration. At four different time points (0, 24, 48, and 72h), Cell Counting Kit-8 (CCK-8) solution was added into each well and incubated for 2hrs. The plate was read at 450 nm for determining the number of viable cells.

### Colony formation assays

Lovo cells were seeded into 12-well plates and allowed to grow for about 4 to 5 days at 37°C until small colonies could be detected. Cell culture plates containing colonies were gently washed with phosphate-buffered saline solution and fixed with 3.7% formaldehyde for 10 min. Finally, the number of colonies was recorded.

### Statistical analysis

All data were analyzed with SPSS 19.0 software (Shanghai, China) through the X^2^ and Student t-test. Values were expressed as the mean±standard deviation (SD). P < 0.05 was considered to be statistically significant.

### Ethical statement

The study protocol was approved by the committee for the Ethical Issue in First People’s Hospital, Shanghai Jiaotong University (No.201304), and written informed consent was obtained from each patient. All methods were conducted in accordance with the Guidelines for Experiments of Shanghai Jiaotong University.

## Results

### *HspB5* over-expression in CRC patients

**Fig 1:**
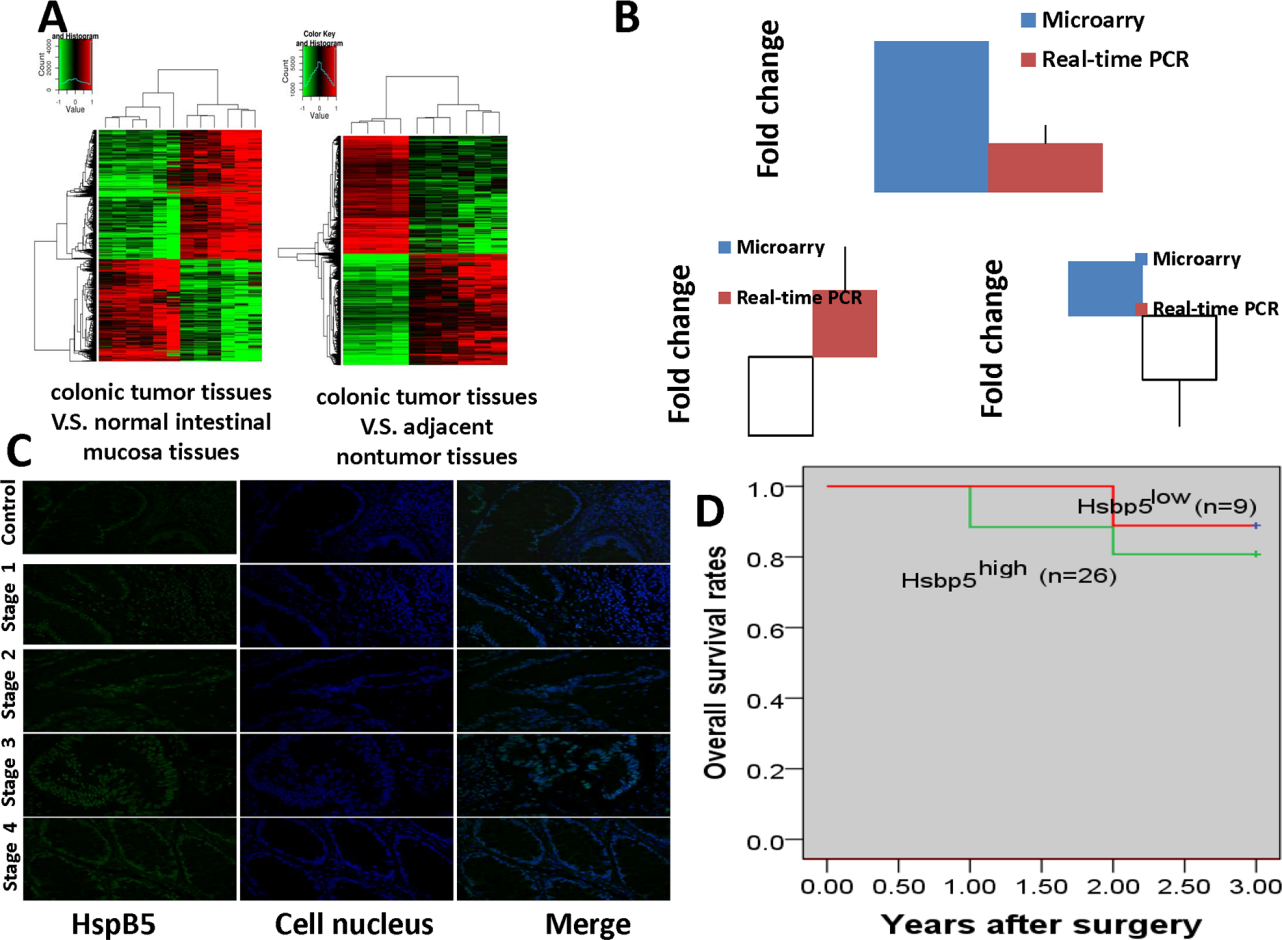
*HspB5* over-expression correlated with CRC patients. A:Hierarchical clustering of gene expression in CRC patients (N = 6). Red and green indicated high and low relative level, respectively. B: A comparison between qRT-PCR (N = 70) and gene microarray assay (N = 6) in CRC patients. The groups were divided into two groups, colonic tumor tissue and their adjacent nontumor tissue. qRT-PCR showed the up-regulation of *HspB5* and MMP7, and down-regulation of E-cadherin in CRC patients. C: Immunofluorescence staining of *HspB5* expression in CRC patients. Green fluorescence and blue fluorescence respectively represents *HspB5* and the cellular nucleus. Stage 1, T1(mucosa); Stage 2, T2(muscle); Stage 3, T3(serosa); Stage 4, T4(viscera). D:*HspB5* indicates poor survival in colorectal cancer during a 3-year follow up (N = 35).CRC patients with high *HspB5*expression had a shorter overall survival rate than those with low expression levels.

There was a significant difference in gene expression among CRC patients. When compared to healthy people, CRC patients were associated with the up-regulation of 1871genes and down-regulation of 1478 genes. In addition, colonic tumor specimens and adjacent nontumor tissue from each CRC patient was collected to further detect the differentially expressed genes. Between these two groups, 1823 genes were differentially expressed in tumor tissue. Of these, 1018 genes were up-regulated and 805 were down-regulated. Hierarchical clustering of gene expressions is shown in Fig 1A.

The microarray gene expression data analysis of the three significant tumor genes is summarized in Table 2. *HspB5*expression in colonic tumor tissue was significantly lower than that in both adjacent nontumor tissue and normal intestinal mucosa samples (P<0.05). High MMP7 expression was found in tumor tissue while E-cadherin expression was not altered (P>0.05). This result showed that there was a significant difference in *HspB5* and MMP7 expression among CRC patients, but not in E-cadherin.

**Table 2:** Gene microarray results for differentially expressed tumor genes in CRC patients.

Gene name	P	Fold
Hsp5B(tumor: adjacent nontumor)	0.033409	0.031731
Hsp5B(tumor: normal tissues)	0.006775	0.632249
MMP7(tumor: adjacent nontumor)	0.000114	243.7458
MMP7(tumor: normal tissues)	0.000275	260.6385
E-cadherin (tumor: adjacent nontumor)	0.377876	1.169679
E-cadherin (tumor: normal tissues)	0.432123	0.897454

To validate the microarray results, 70 CRC patients and 40 healthy people were chosen and sampled material subjected to the further study using qRT-PCR. As seen in Table 3, *HspB5*, MMP7 and E-cadherin were validated to be differentially expressed when comparing colonic tumor tissue and adjacent nontumor samples from CRC patients. Contrary to the down-regulation of *HspB5* found in gene microarray results, *HspB5*in colonic tumor tissue was significantly higher at mRNA level (colonic tumor tissue vs. adjacent nontumor tissue, P<0.05). The same result was found in CRC patients vs. healthy controls. E-cadherin in qRT-PCR analysis was also validated to be significantly down-regulated among these CRC tissue samples (P<0.05) while it did not show an obvious change when measured using gene microarray. MMP7 was over-expression in colonic tumor tissue (P<0.05), showing the same results from both qRT-PCR and gene microarray analysis. A comparison between qRT-PCR and gene microarray is described in Fig 1B. In summary, qRT-PCR analysis confirmed three differently expressed genes in CRC patients which included up-regulation of *HspB5* and MMP7, and down-regulation of E-cadherin.

**Table 3:** qRT-PCR confirmations of 3 differentially expressed genes among 70 CRC patients.

Gene name	2^-ΔΔCt^Regulation			Regulation
	tumor tissue	adjacent nontumor tissue	normal tissue	
Hsp5B	24.9±16.6	12.6±6.6	7.9±3.5	Up
MMP	29.7±78.1	1	-	Up
E-Cadherin	0.7±0.5	1	-	Down

*HspB5*-labeled fluorescence experiments demonstrated that tumor tissue from CRC patients exhibited a stronger green fluorescence than their normal controls, and *HspB5* labeled green fluorescence was found near the nucleus of the cell (Fig 1C). The experiments clearly revealed that *HspB5* levels had markedly increased in CRC patients at the protein level (stage 4>stage 3>stage 2>stage 1>normal intestinal mucosa tissue), which is consistent with TNM descriptors.

### *HspB5* over-expression correlates with clinicopathological parameters and poor prognosis in CRC patients

The relationship between *HspB5* over-expression and clinicopathological characteristics in CRC patients is shown in Table 4. The groups with high *HspB5* expression levels accounted for 61.4% (43/70) of the total number of patients. High *HspB5* expression was associated with TNM staging in 70 CRC patients (P<0.05). However, other important clinical characteristics, including age, sex, tumor size, tumor differentiation, lymph node metastasis, and tumor location were not directly related to *HspB5* expression.

**Table 4:** Correlation between *HspB5* and clinicopathological characteristics in 70 CRC patients.

Variables	N	Hsp5B		P
		High	Low	
Patient	70	43	27	
Sex				0.776
Male	44	34	10	
Female	26	19	7	
Tumor diameter (cm)				0.144
≥5cm	25	16	9	
<5cm	45	37	8	
Location				0.248
Colon	27	18	9	
Rectum	43	34	9	
TNM stage				0.042
Ⅰ	3	3	0	
Ⅱ	14	7	7	
Ⅲ	20	18	2	
Ⅳ	33	25	8	
Lymphatic metastasis				0.584
Yes	32	24	8	
No	38	29	9	
Distant metastasis				0.622
Yes	9	7	2	
No	61	46	15	
Tumor differentiation				0.084
Low	18	13	5	
Middle	49	38	11	
High	3	2	1	

Patient follow-up was terminated on May 15, 2016, with the follow-up period for each of the patients ranging from 12 to 48 months. In order to analyze the overall survival rate, data from three year post-operative follow-up visits of 35 patients was used. Fig 1D reveals that the patients with high levels of *HspB5* had a shorter overall survival rate than those with low expression. More patient samples will be collected to analyze the survival rates in future studies.

### *HspB5* promotes cell proliferation and invasion

**Fig 2:**
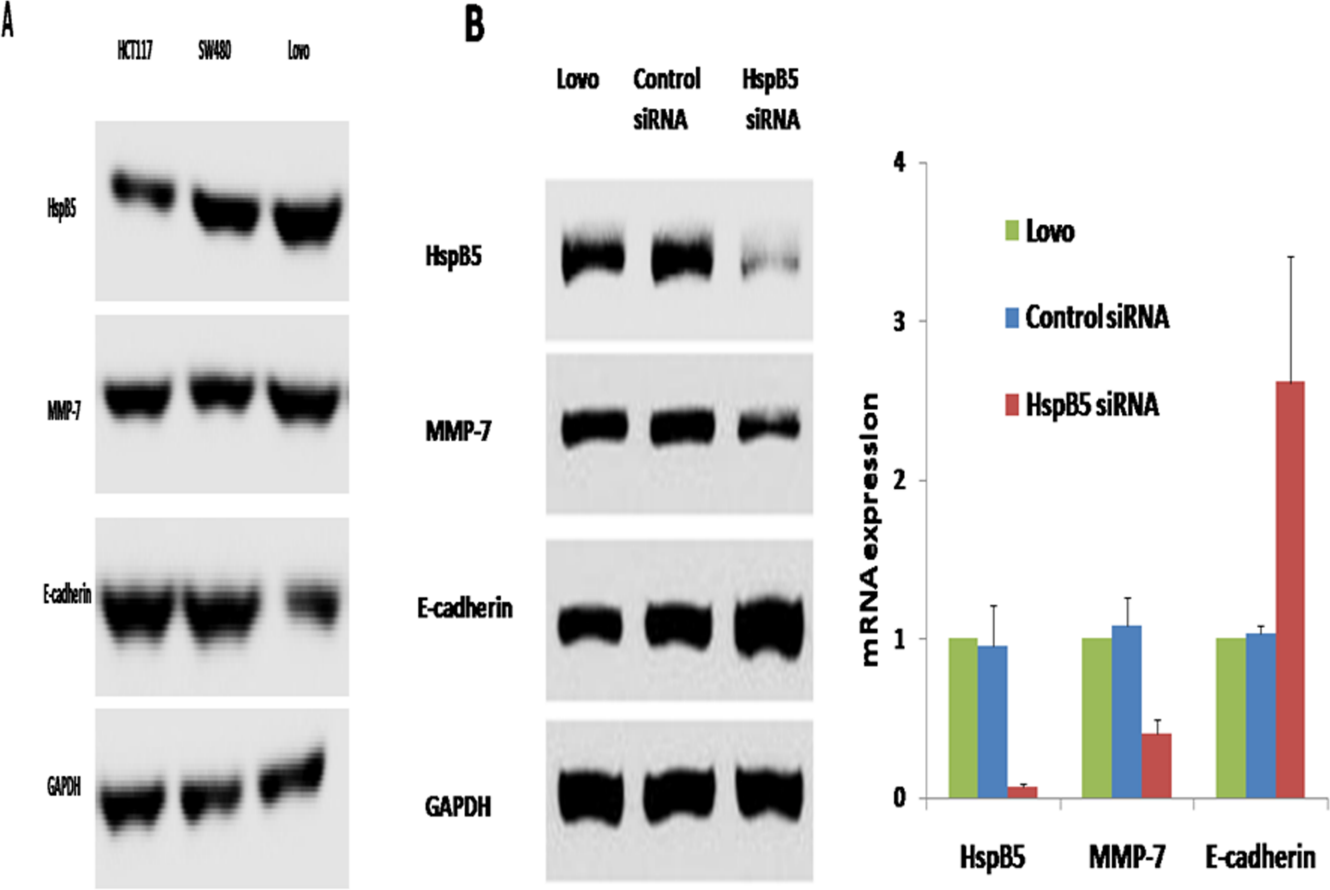
*HspB5* over-expression in Lovo cells and treated by siRNA. A: Western blot. There has been a significant increase in *HspB5* expression in Lovo cells, but not in HCT117 and SW480. B: Western blot and qRT-PCR analysis in Lovo cells treated by siRNA for 6 h. There was a significant decrease in *HspB5* expression in the *HspB5* siRNA-treated group. The control siRNA group failed to reveal any differences in *HspB5* expression before and after *HspB5* siRNA interference.

Three different CRC cell lines, HCT116, SW480 and Lovo cells, were considered as the *in vitro* cellular model in this study. Western blot analysis showed that *HspB5* protein levels in Lovo cells were significantly higher than those in HCT117 and SW480 cells (Fig 2A). *HspB5* mRNA in Lovo cells was also highly expressed compared to the other two cell lines. Thus, Lovo cells were chosen to determine the molecular mechanism of *HspB5* in CRC.

siRNAs were used to reduce the levels of *HspB5* expression in Lovo cells. As seen in Fig 2B, western blot analysis showed that there was a 90% reduction of *HspB5* protein levels after siRNA-mediated gene knockdown. Fig 2B showed that *HspB5* mRNA levels were significantly lower after the introduction of *HspB5* siRNA (0.07±0.02 vs.1, P<0.01). The control siRNA group failed to reveal any differences in *HspB5* expression before and after siRNA interference. These data clearly revealed that gene silencing with siRNA successfully knocked out the *HspB5* expression in Lovo cells, which could serve as the *in vitro* model to investigate the tumor invasion and metastasis in CRC.

**Fig 3:**
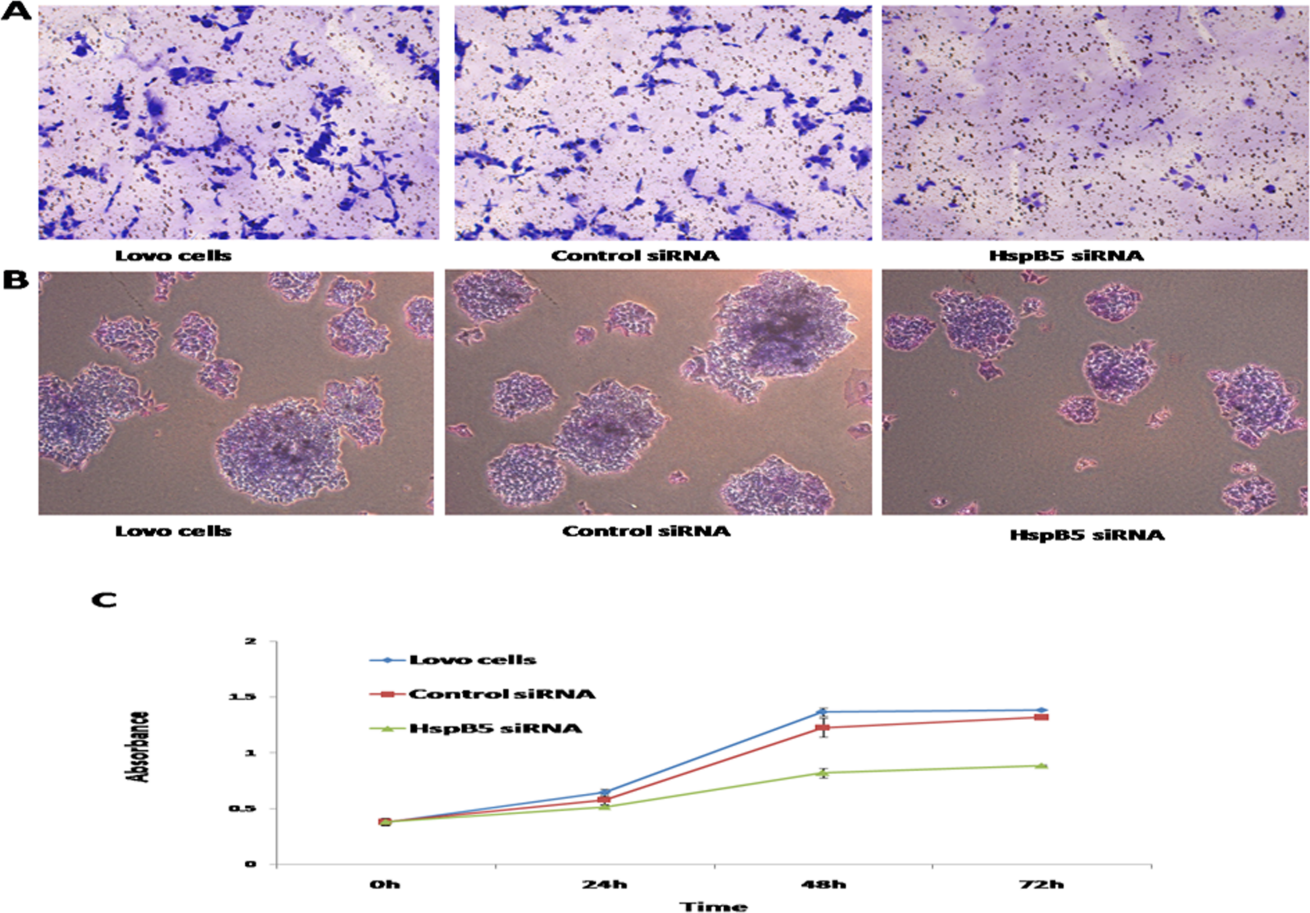
*HspB5* promotes cell proliferation, invasion and colon formation. A:Transwell Matrigel invasion assays. The number of invasive cells in the *HspB5* siRNA-treated group decreased obviously compared with those in the control group.B: In colony forming assay, cell proliferation was significantly suppressed by *HspB5* siRNA and ERK inhibitor (N = 3). Lovo cells vs.*HspB5* siRNA(5.0±0.5%vs. 4.3±0.3%). C: CCK-8 assay. The cell proliferation was significantly suppressed 48 hours after *HspB5* siRNA transfection. *HspB5* over-expression could prompt tumor cell proliferation in CRC.

There were obvious signs of tumor cell proliferation and invasion in Lovo cells, as seen in Fig 3. After siRNA-*HspB5* treatment for 6 h, the number of invasive cells decreased markedly along with the inhibition of *HspB5* expression (Fig 3A). The colony forming assay (Fig 3B) and CCK-8 assay (Fig 3C) revealed that Lovo cell proliferation was significantly suppressed by *HspB5* siRNA. In addition, the control siRNA group failed to reveal any differences compared to Lovo cells. Based on this data, this study clearly shows that *HspB5* over-expression could prompt the ability of tumor cell proliferation and invasion in CRC.

### *HspB5* induces CRC EMT *in vivo* and *in vitro*

To investigate the effect of *HspB5* on inducing EMT, the expression of two EMT hallmarks, E-cadherin and MMP7, were assessed *in vivo* and *in vitro*. Our clinical data indicated that *HspB5* was up-regulatedin CRC patients. Accompanied by *HspB5* over-expression, up-regulation of MMP7 and down-regulation of E-cadherin were found in CRC patients (Fig 1B). This implies that elevated *HspB5* expression induces EMT in CRC. After *HspB5* gene silencing with siRNA, up-regulation of E-cadherin expression and down-regulation of MMP7 expression were founded in Lovo cells (Fig 2B). The *in vitro* study further established that the increased expression of *HspB5* induces EMT in colorectal cancer.

### Hyperactivation of ERK signaling is responsible for *HspB5*-induced EMT

**Fig 4:**
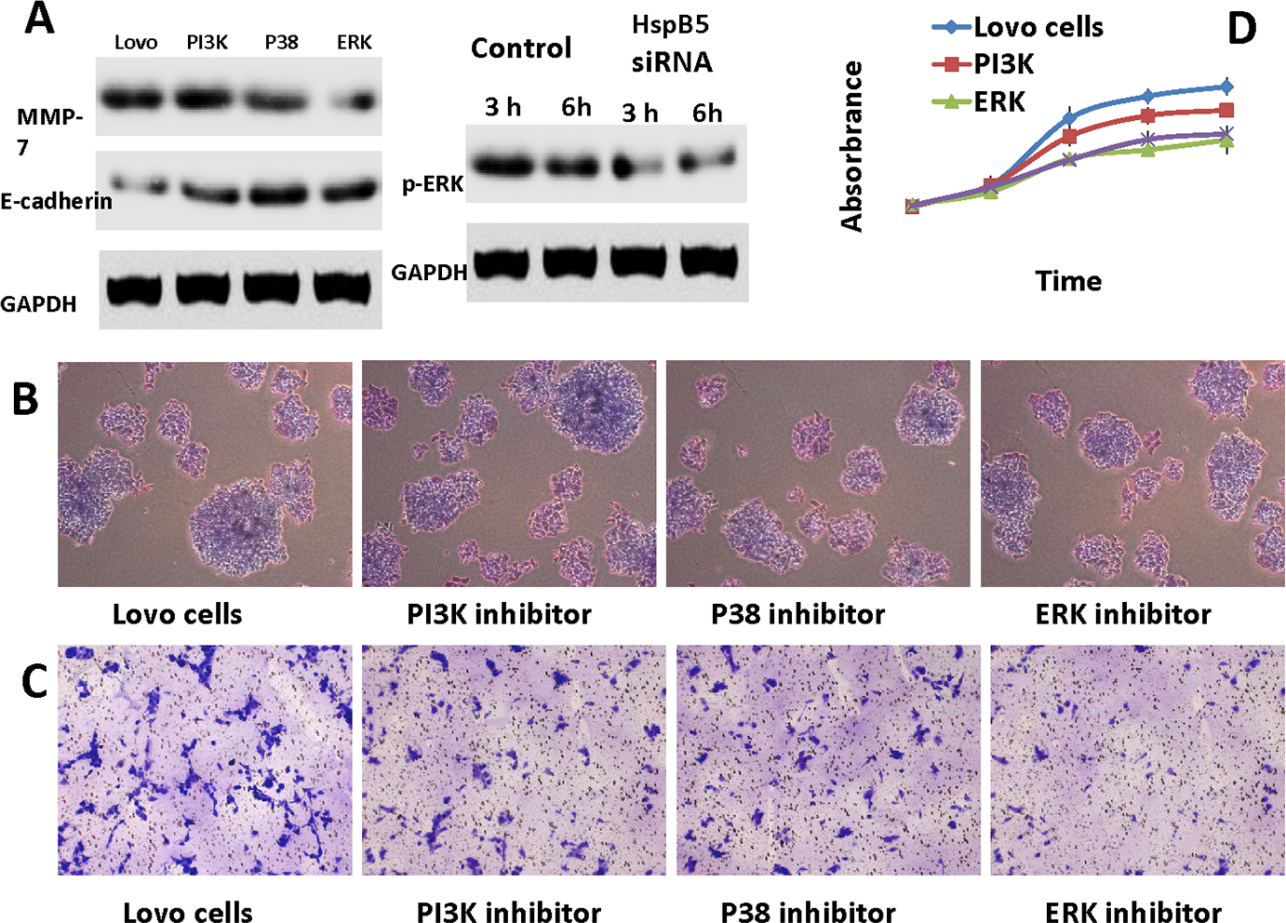
*HspB5* promotes the EMT process via the ERK signaling pathway. A: Western blot analysis. There has been a significant decrease inMMP7, and an obvious increase in E-cadherin in Lovo cells treated by an inhibitor of ERK. An obvious down-regulation of ERK treated by siRNA-*HspB5*was found. B: In colony forming assays, cell proliferation was significantly suppressed by an ERK inhibitor (N = 3). Lovo cells vs. ERK inhibitor(5.0±0.5%vs. 3.2±0.3%). C: Transwell Matrigel invasion assays. The number of invasive cells was obviously inhibited in the ERK-treated group compared to the control group. D: CCK-8 assay using a pathway inhibitor. Cell proliferation was significantly suppressed through an inhibitor of the ERK pathway.

Using the Kyoto Encyclopedia of Genes and Genomes and Pathway Profiler databases, the three significant signaling pathways, phosphatidylinositol 3-kinase(PI3K), p38 mitogen activated protein kinase (p38MAPK) and extracellular regulated protein kinases (ERK), were detected in this study. These pathways have previously been reported in metastasis and carcinogenesis [13]. To further explore the role of the signaling pathways in *HspB5*-induced EMT, Ly294002 (PI3K inhibitor), SB202190 (p38 inhibitor) and PD98039 (ERK inhibitor) were used to treat Lovo cells. As shown in Fig 4A, down-regulation of MMP7 and up-regulation of E-cadherin at protein level in ERK inhibitor-treated groups was observed, but not in PI3K and p38 inhibitor groups. The latter two groups indicated a typical EMT phenotype due to the hyperactivation of ERK. Furthermore, cellular invasion and proliferation assays were used to investigate the cellular characteristics. After blocking ERK, PI3K and p38signaling, cellular invasion and proliferation assays were evidently inhibited by the ERK signaling pathway (Fig 4B–4D). The aforementioned data clearly revealed that the ERK signaling pathway may be crucially involved in invasion, proliferation and EMT. Finally, siRNA-*HspB5* was used to treat Lovo cells at 3 and 6h. This result indicated that the ERK signaling pathway was significantly blocked after *HspB5* knockdown, thereby showing that *HspB5* could regulate ERK (Fig 4A). Taken together, these results suggest a possible mechanism by which *HspB5* induces EMT by activating ERK signaling in CRC cells.

## Discussion

In this study, we explored the clinical significance and underlying mechanism of *HspB5* in CRC pathogenesis. First, expression of *HspB5* was significantly increased in CRC tissue compared with that in adjacent non-tumor or normal intestinal mucosa tissues. Second, *HspB5* over-expression was closely correlated with the TNM stage and poor prognosis in clinical practice. This is the first report on the molecular mechanism of *HspB5* and its involvement in tumor invasion and metastasis in CRC. This study clearly indicates that *HspB5* is a candidate tumor suppressor which was markedly up-regulated in CRC, and potently induces the EMT process by activating the ERK signaling pathway.

The gene expression profiles chip is the latest DNA analysis technology using a whole-genome expression design[17]. It has been widely applied to screen the differentially expressed genes as potential biomarkers in cancer patients. With the great genetic scale, high throughput and objectivity, it allows the monitoring of tens of thousands of genes from each patient. As reported previously, *HspB5* aggravated the oncogenic transformation[18]. This is achieved by involving extensive regulatory functions in cell apoptosis, proliferation, migration, invasion, drug resistance, and cell cycle regulation through inhibiting caspase-3 activation[19, 20], and anti-VEGF (vascular endothelial growth factor) resistance[5].

In this study, we found that *HspB5* was obviously up-regulated in qRT-PCR while *HspB5* was down-regulated according to gene microarray results. In order to explain the contradiction, the five samples analyzed with the gene chip were validated using qRT-PCR. The over-regulation of *HspB5* mRNA of three samples and down-regulation of *HspB5* mRNA of two samples was confirmed. We further investigated the level of *HspB5* mRNA expressionof70 CRC patients, in which 43 *HspB5* mRNA cases were up-regulated and 27 *HspB5* mRNA cases were down-regulated. In addition, E-Cadherin in qRT-PCR was down-regulated while it had not an obvious difference in gene microarray. To the best of our knowledge, it was impossible that gene expression in tumor patients was up to 100% positive rate due to the individual differences[21]. Thus, the discrepancies in the results of qRT-PCR and gene microarray analysis could be attributed to the individual differences in clinical patients. It is necessary that qRT-PCR is performed to validate the microarray results.

To date, *HspB5* has been reported to be over-expressed in the multiple tumors, and is associated with poor prognosis and recurrence of human cancer. In this study, *HspB5* over-expression was founded in CRC patients. This is consistent with other types of cancers, such as breast, thyroid, renal papillary cell, gastric, oral, and hepatocellular carcinoma[8, 22, 23]. Results from previous studies suggested that *HspB5* over-expression may play an important role in CRC. Subsequently, we studied the relationship between *HspB5* over-expression and poor prognosis in CRC patients. Histopathological characteristics, such as the TNM stage, larger tumor size, presence of vascular invasion, intrahepatic spreading, and lymph nodemetastasis, were regarded as the significant hallmarks of poor prognosis in CRC patients[24]. *HspB5* expression was found to be up-regulated with TNM stage cancer (P = 0.042), indicating that *HspB5* could be regarded as a novel biomarker for CRC prognosis in such patients.

Since CRC cells undergoing EMT is a critical initiation event for tumor progression, their detection and identification will aid in the development of new therapies that specifically target EMT, improve patient prognosis, and reduce therapy resistance[25]. Playing a key role in cellular proliferation, invasion and metastasis, *HspB5* was responsible for the poor prognosis in *HspB5*-positive cancer patients[26]. *HspB5* was validated as a potent inducer of EMT, an important mechanism during cancer invasion transformation and metastasis. However, *HspB5*-induced EMT in CRC was unknown. We firstly demonstrated that *HspB5* expression could be inhibited by siRNAs in Lovo cells. After blocking *HspB5* expression, the ability of tumor cell proliferation, invasion and metastasis in CRC was dramatically reduced. E-cadherin and MMP7 [27] are thought to promote tumor metastasis and progression in a variety of human cancers by inducing EMT. Changes in E-cadherin levels were related to the adhesion of epithelial cells, allowing cancer cells to cross the basement membrane and invade surrounding tissue. MMP7 plays an important role in tumor metastasis and regulation of cell migration through the breakdown of the extracellular matrix. Next, our study illustrated the down-regulation of E-cadherin and up-regulation of MMP7 *in vivo* and *in vitro*, especially in CRC patients. Furthermore, a recent paper reported that *HspB5* promoted tumor migration and invasion capability through EMT signaling in CRC SW480 cell and nude mice model[28].Thus, these data revealed that CRC cells had undergone an EMT process that was identified as a result of the increase in *HspB5* proteins.

Out of further interest, we investigated the possible pathway by which *HspB5* participates in cell metastasis of colorectal carcinoma. We found that the inhibitor of ERK, but not PI3K/Akt or p38, attenuated the EMT progression in CRC. We demonstrated that PD98039, which serves as an ERK inhibitor, successfully up-regulated the level of E-cadherin, and down-regulated the level of MMP7 *in vitro*, leading directly to decreased migration and invasion in tumor cells. A study had indicated that the PI3K/Akt, ERK and Raf/MAPK signaling pathways played an important role in the process of EMT inhuman cancers[29]. In theory, the up-regulation of *HspB5* expression would participate in PI3K, p38 and ERK signaling in CRC cells[30–32]. Bewilderingly, the ERK, but not PI3K and p38 signaling pathways may be crucially involved in the invasion, proliferation and EMT induced by *HspB5* over-expression in Lovo Cells. One plausible explanation is that the functions of PI3K/Akt, ERK and Raf/MAPK in EMT might depend on the different cell types[33].

## Conclusion

In a word, this study provides a better understanding on both the functional role and molecular mechanism of *HspB5* in human CRC. Our current work indicates that *HspB5* is a novel marker for the unfavorable prognosis in CRC patients after surgery due to its capacity to facilitate cancer cell migration and invasion. Notably, *HspB5* may induce EMT via the ERK signaling pathway in CRC.
